# Integrating Drug Physicochemistry and Hfq-Dependent Regulation of Ciprofloxacin Accumulation in *Escherichia coli*

**DOI:** 10.3390/pathogens15101082

**Published:** 2026-10-15

**Authors:** Gabriela Mistygacz, Florian Turbant, Blandine Pineau, Aleksandra Łukasiak, Marisela Velez, Frank Wien, Grzegorz Węgrzyn, Sylwia Bloch, Véronique Arluison

**Affiliations:** 1 Laboratoire Léon Brillouin LLB, UMR12 CEA CNRS, CEA Saclay, 91191 Gif-sur-Yvette, France; gabriela.mistygacz@phdstud.ug.edu.pl (G.M.); turbantf@ext.ill.fr (F.T.); 2 Department of Molecular Biology, Faculty of Biology, University of Gdansk, Wita Stwosza 59, 80-308 Gdansk, Poland; aleksandra.lukasiak@phdstud.ug.edu.pl (A.Ł.); grzegorz.wegrzyn@ug.edu.pl (G.W.); sylwia.bloch@ug.edu.pl (S.B.); 3 Synchrotron SOLEIL, L’Orme des Merisiers, Saint Aubin BP48, 91192 Gif-sur-Yvette, France; blandine.pineau@synchrotron-soleil.fr (B.P.); frank.wien@synchrotron-soleil.fr (F.W.); 4 Université Grenoble Alpes, CNRS, LiPhy, 38000 Grenoble, France; 5 Instituto de Catálisis y Petroleoquímica, CSIC, c/Marie Curie, 2, Cantoblanco, E-28049 Madrid, Spain; marisela.velez@icp.csic.es; 6 UFR SDV, Université Paris Cité, 75013 Paris, France

**Keywords:** antimicrobial resistance, antibiotic influx, fluoroquinolones, pH homeostasis, Hfq, outer membrane porins, small non-coding RNA, multilevel regulation, membrane permeability

## Abstract

Ciprofloxacin (CIP), an amphoteric fluoroquinolone, undergoes pH-dependent changes in protonation state that influence membrane permeability and intracellular accumulation. Here, we investigated how drug physicochemistry and bacterial regulatory pathways jointly determine CIP accumulation in *Escherichia coli* using single-cell fluorescence measurements under controlled extracellular pH conditions. Mutants with *hfq* deletion and C-terminally truncated Hfq variants (CTR), as well as Δ*ompC* and Δ*ompF* mutants, were used. At pH 5, ∆*ompC*, ∆*ompC* ∆*ompF*, and ∆*hfq* strains all exhibited similarly low CIP accumulation, consistent with the limited penetration of CIP through OmpC and OmpF porins under such conditions. Importantly, we observed an increased CIP influx in ∆*ctr* compared to wild-type bacteria. At neutral pH 7, overall CIP accumulation increased relative to acidic conditions; however, the relative trends among the mutants remained similar. At pH 9, low efficiency of CIP accumulation occurred in the ∆*ompC* ∆*ompF* double mutant. When measuring intracellular pH, wild-type cells maintained a higher cytoplasmic pH value, ~7.4, when compared to ∆*hfq*, ∆*ompC*, and ∆*ompC* ∆*ompF* strains, which exhibited similar, lower pH values ~7.2. Atomic force microscopic studies revealed that both the Hfq-CTR and the OmpC fragment (a peptide consisting of residues 220–237) formed fibrillar structures when examined separately, but when the two peptides were combined, the resulting structures displayed an architecture distinct from that observed for either peptide alone. Together, these findings suggest a possible contribution of Hfq-modulated pH homeostasis to altered CIP accumulation.

## 1. Introduction

Antibiotics have saved millions of lives and revolutionized the treatment of infectious diseases. However, the emergence and spread of multidrug-resistant bacteria (MDR) have triggered an “antibiotic crisis,” threatening a return to the pre-antibiotic era if new strategies are not developed [1]. Beyond the search for novel antimicrobial agents, a detailed understanding of the genetic and molecular mechanisms underlying bacterial susceptibility and resistance is essential for improving therapeutic outcomes. Among the key antimicrobial classes currently in clinical use are fluoroquinolones, broad-spectrum antibiotics that target DNA gyrase and topoisomerase IV [2,3]. Bacterial resistance to these drugs typically arises from mutations affecting the target enzymes, multidrug efflux pumps, or outer membrane (OM) porins that mediate drug passage into the cell [4,5].

Fluoroquinolones are small amphoteric molecules (typically 300–400 Da) containing both a deprotonatable carboxylic acid and a protonatable amine, resulting in pH-dependent speciation into cationic (pH < 5), zwitterionic (pH ~6–8), and anionic forms (pH > 9) [6]. Ciprofloxacin (CIP), a widely used fluoroquinolone, exemplifies this behavior through its carboxylate and piperazinyl groups [6]. These protonation equilibria strongly influence the hydrophobicity and membrane permeability of CIP [7]. To reach its intracellular targets, CIP must cross both the outer and inner/cytoplasmic membranes of Gram-negative bacteria, whereas the cell wall is thought to provide little or no barrier to diffusion. In *Escherichia coli*, CIP uptake is strongly influenced by pH-dependent changes in both porin permeability and antibiotic charge, thereby affecting intracellular accumulation and antibacterial activity [8,9].

The ionization state of CIP strongly influences its membrane permeability. At near-neutral pH, CIP predominantly exists in a zwitterionic or weakly charged form, favoring membrane translocation [10]. Under alkaline conditions, CIP becomes increasingly anionic, reducing its passive permeability across biological membranes [10]. Conversely, under acidic conditions, CIP is predominantly cationic, which also decreases its lipophilicity and limits diffusion through the lipid bilayer [10]. Changes in protonation state therefore affect the pathways through which CIP traverses the bacterial envelope. Under neutral or mildly alkaline conditions, CIP primarily enters through outer membrane porins [11,12], with OmpF generally supporting greater fluoroquinolone flux than the narrower OmpC channel [13]. In contrast, extracellular acidification reduces outer membrane permeability by increasing the closing probability of OmpF and OmpC through protonation of residues within their constriction zones, thereby decreasing pore conductance [13,14]. At the same time, the predominance of the cationic form of CIP decreases its lipophilicity and limits passive diffusion across lipid bilayers [10].

Extracellular pH also remodels the expression and activity of proteins involved in CIP accumulation. At neutral pH, OmpC and OmpF are produced at comparable levels [15,16], while efflux systems remain active [17,18]. Acidic conditions alter porin expression through the EnvZ–OmpR two-component regulatory system [19], promoting *ompC* transcription [20,21], while repressing *ompF* translation through the MicF small noncoding RNA (sRNA) [22,23]. Low pH also reduces the activity of multidrug efflux systems, including AcrB–TolC [24]. Under alkaline conditions, by contrast, *ompF* expression is favored relative to *ompC* [15], and CIP influx is expected to rely predominantly on the wider OmpF channel [13]. Because both porin channels favor cation transport [7], negatively charged quinolones can chelate Mg^2+^ ions at the outer membrane surface, generating Mg^2+^–quinolone complexes with a net positive charge that can traverse the porin. Meanwhile, active efflux contributes to CIP removal [17,18,24]. After entering the periplasm, CIP must still cross the inner membrane to reach its cytoplasmic targets. Because the periplasmic pH is typically close to neutral (6.8–7.0 under standard conditions) [25], fluoroquinolones can cross the inner membrane predominantly by diffusion. The more alkaline cytoplasmic pH (~7.4) may then promote intracellular accumulation through ion trapping of the slightly negatively charged form of CIP [4,25].

Recent findings have added an unexpected component to this picture by implicating the RNA chaperone Hfq in the *E. coli* cellular response to fluoroquinolones [26]. Hfq is an RNA-binding protein that functions as a global post-transcriptional regulator by facilitating interactions between small regulatory RNAs (sRNAs) and their target mRNAs [27]. Through these interactions, Hfq modulates mRNA stability and translation [28], enabling rapid adaptation of gene expression to environmental and cellular stress [29]. Hfq regulates translation primarily by altering the accessibility of the ribosome-binding site (RBS) on target mRNAs. By simultaneously binding sRNAs and target mRNAs, Hfq promotes sRNA–mRNA base pairing or remodels RNA secondary structures [30]. When bound to mRNAs near the RBS, Hfq can affect ribosome loading while coupling translational regulation to mRNA stability [31]. In most cases, Hfq acts as a negative regulator by facilitating sRNA-mediated repression of target mRNAs. However, notable exceptions include *rpoS*, encoding the alternative sigma factor σ^S^, and *gadX*, encoding a regulator involved in acid resistance, for which Hfq positively regulates translation [32,33].

The N-terminal region (NTR) of Hfq forms a hexameric torus primarily responsible for RNA binding and annealing [31,34,35,36]. The C-terminal region (CTR), once considered intrinsically disordered, can adopt a β-rich, amyloid-like conformation [37]. Although dispensable for most sRNA-mediated regulatory pathways [38], Hfq-CTR contributes to a subset of specific sRNA-dependent regulatory processes [39,40,41]. For example, Hfq lacking its CTR is defective in DsrA sRNA-mediated enhancement of *rpoS* mRNA translation, encoding the sigma factor σ^S^ [42,43], as well as in RybB-mediated regulation of multiple outer membrane protein mRNAs, including *ompC* and *ompF* transcripts [44]. Notably, σ^E^-regulated sRNAs are particularly sensitive to Hfq-CTR deletion [41].

Hfq participates in diverse cellular processes, including bacterial susceptibility to antibiotics [45,46,47,48]. Recent studies involving fluoroquinolones indicate that Hfq influences CIP accumulation through a complex regulatory network that may involve both AcrB-mediated efflux and Omp-dependent membrane permeability, with contributions from sRNA-dependent and sRNA-independent mechanisms [26,49]. One proposed sRNA-independent mechanism involves direct interactions between Hfq and bacterial membranes. In particular, the Hfq-CTR has been reported to induce pore formation and alter inner and outer membrane structures [50,51,52,53]. Consistent with this possibility, deletion of either the entire *hfq* gene or the region encoding its CTR significantly altered CIP accumulation even in Δ*ompC*/Δ*ompF* mutants [26,49]. These observations suggest that Hfq can influence fluoroquinolone accumulation through mechanisms that are at least partly independent of the major OmpC and OmpF porins and are consistent with proposals that Hfq may modulate membrane properties or efflux processes [51]. Because fluoroquinolones can cross the outer membrane through both porin-dependent and lipid-mediated routes depending on their protonation state, Hfq-dependent alterations in pH homeostasis, sRNA-mediated regulation of membrane composition, or bilayer permeability could contribute to this phenotype [54]. Although Hfq may also contribute to the regulatory network through post-transcriptional regulation of *acrB* [55]; its effect on CIP accumulation appeared to be largely independent of AcrB-mediated efflux and is instead thought to primarily involve modulation of outer membrane permeability [26].

In this study, we investigated the interplay between extracellular pH and allelic variations in *hfq*, *ompC*, and *ompF* on CIP accumulation in *E. coli* to elucidate the contribution of Hfq to fluoroquinolone uptake and the cellular mechanisms underlying its effect on CIP accumulation.

## 2. Materials and Methods

### 2.1. Bacterial Strains

*E. coli* strains used in this study are listed in Table 1. All are derivatives of the *E. coli* AG100 strain.

A series of isogenic AG100 strains bearing mutations in *ompC* and *ompF* genes were constructed using standard P1 transduction [58] and were described previously [49]. The AG100 derivatives devoid of the entire Hfq protein (∆*hfq*::*cm*) or producing its truncated form with only the first 72 amino acid residues, corresponding to the N-terminal domain of Hfq (Δ*ctr* or *hfq*72-*cm*), were previously described [26,49]. Mutant strains were grown at 37 °C in LB medium supplemented with chloramphenicol (25 µg/mL) when necessary.

### 2.2. Assessment of Intracellular Ciprofloxacin Accumulation

To quantify drug influx, a single-cell CIP fluorescence assay was used [59]. CIP (Sigma-Aldrich, Burlington, MA, USA) accumulation was measured at the single-cell level using the deep-UV fluorescence inverted microscope (TELEMOS) on the DISCO beamline at SOLEIL synchrotron (proposal 20242209). The micro-spectrofluorimetry experiments were performed as described previously [26,49]. Briefly, cells (cultures at OD_600_ = 0.6) were concentrated 10-fold in NaPi-Mg sodium phosphate buffer (50 mM sodium phosphate with 0.5 mM MgCl_2_ at pH 5, 7 or 9). When present, CIP was added at 10 µM. Cells were placed between quartz coverslips for analysis. For quantification, a series of acquisitions were performed using an excitation wavelength of λ_exc_ = 275 nm and two emission windows ranging from 329 to 351 nm and from 420 to 480 nm, corresponding to tryptophan (Trp) and CIP fluorescence, respectively, as previously described [26,49]. Images were analyzed using ImageJ 1.54 and the Python-based analysis workflow developed at the DISCO beamline. Individual bacterial cells were analyzed at the single-cell level, and CIP fluorescence signals were normalized to the corresponding intrinsic Trp fluorescence signal, following the procedure reported previously [26,49]. Under each tested condition (bacterial strain and treatment), three biological replicates were performed (three independent cell cultures), and >20 cells were analyzed from each independent experiment as technical replicates. Then, average values from these three repeats were compared, and since no statistically significant differences (*p* > 0.05) between the groups were detected (this was true for all investigated variants of the experiments), the results from all cells were pooled and analyzed. Note that each cell was independently measured only once. Thus, no individual cell was repeatedly sampled or measured, and the same cell was not included more than once in the analysis. Therefore, individual-cell measurements represent independent observations within each biological replicate and not repeated measurements, ensuring that each cell contributed only a single observation to the dataset. Mean values are presented in figures with error bars indicating the standard deviation (SD). The values were compared using the one-way ANOVA followed by Tukey post hoc tests. Levene’s test was used to check the homogeneity of variance assumption. The *p* values < 0.05 were considered statistically significant.

### 2.3. Analysis of Bacterial Cell Growth and Viability During Ciprofloxacin Accumulation at Different pH Ranges

To check the effect of CIP accumulation on bacterial growth and viability at different pH ranges, two different approaches were performed. In the first one, the overnight bacterial culture was diluted (1:500) into LB medium with different pH ranges. Then, the culture was divided into two aliquots. One of them was treated with 10 µM CIP and the second one was a control without antibiotics. In the next step, 0.180 mL of bacterial culture was transferred to a 96-well polystyrene plate and the cultivation was continued at 37 °C for 17 h in the Varioscan Lux plate reader (Thermo Fisher Scientific, Waltham, MA, USA). At indicated times, the density of bacterial cultures, monitored by OD_600_ measurement, was examined.

In the second experimental approach, bacteria were grown in LB medium (pH 7.2) at 37 °C to an OD_600_ of 0.3. Then, 2 mL of bacterial culture was centrifuged (2000× *g*, 20 min, 4 °C) and the pellet was suspended in 2 mL of NaPi-Mg sodium phosphate buffer with pH 5, pH 7 or pH 9. In the next step, the culture was divided into two aliquots. One of them was treated with 10 µM CIP and the second one was a control without antibiotics. The cultivation was continued at room temperature without shaking. At the final time point of the experiment (28 min), the bacterial culture density (OD_600_) and the number of colony-forming units per ml (CFU/mL) were examined. To estimate CFU/mL the 10-fold serial dilutions of samples were performed in 0.85% sodium chloride and 0.01 mL of each dilution was directly spotted onto the surface of the LB agar plate. After overnight incubation at 37 °C, the bacterial survivors were counted, and the CFU/mL was determined.

### 2.4. Assessment of Extracellular and Intracellular pH

BCECF (Bis-CarboxyEthyl CarboxyFluorescein), a ratiometric fluorescent pH indicator, was used to assess intracellular pH. BCECF exhibits a fluorescence emission response within the physiological pH window of 6.4–8.0. For cellular uptake, the membrane-permeant acetoxymethyl ester derivative BCECF-AM (Sigma-Aldrich) was employed at 100 µM. Single-cell pH measurements were obtained following an excitation at wavelengths between ~440 and 500 nm, enabling pH-dependent spectral change measurements [60]. Fluorescence was detected through a 535–607 nm emission filter. This configuration enabled an estimation of intracellular pH dynamics at the single-cell level. Note that we report here fluorescence intensity with excitation in the 440–500 nm range, and not the ratiometric readout (F490/F440). This choice was dictated by the need to simultaneously evaluate both pH and CIP accumulation (normalized to the Trp signal) in the same single cell. Unfortunately, the current SOLEIL/TELEMOS configuration does not permit precise excitation of the probe at 440 and 490 nm, restricting excitation to the broader 440–500 nm range. Consequently, the measured values should be regarded as approximate pH estimates rather than accurate quantitative determinations.

Experimentally, the individual bacteria were the same as those analyzed for the single-cell ciprofloxacin fluorescence assay. They were washed three times with NaPi-Mg and resuspended in the same buffer in the presence of pH probe. To test the effect of pH changes on intracellular pH, we used only NaPi-Mg at pH 5 and pH 7. Due to the hydrolysis of BCECF-AM at basic pH, the same analysis was not possible at pH 9. BCECF-AM typically enters the cell within a few minutes. Fluorescence was then recorded after 15 min of incubation to ensure sufficient dye accumulation in cells. The relative fluorescence intensity inside bacteria and in the surrounding region was quantified using ImageJ [61]. The pH in the immediate bacterial environment was assessed under the various buffer conditions to ensure that the expected pH values were maintained throughout the experiments. Measurements were calibrated using cell-free buffers spanning a range of pH values within the interval suitable for accurate quantification, ensuring reliable and consistent pH determination across all conditions.

### 2.5. Assessment of OmpC Self-Assembly Probed by Synchrotron Radiation Circular Dichroism (SRCD), Fourier Transform Infrared (FTIR) and Atomic Force Microscopy (AFM)

Hfq-CTR (residues 64 to 102 of full-length Hfq), and OmpC peptides were chemically synthesized (Proteogenix/France). They were prepared at 20 mg/mL in water and analyzed after 2 weeks. Hfq-CTR sequence was: SRPVSHHSNNAGGGTSSNYHHGSSAQNTSAQQDSEETE; OmpC peptides sequences were: (i) OmpC_52-72_: GYGQWEYQIQGNSAENENNSW, (ii) OmpC_220-252_: GGLKYDANNIYLAAQYTQTYNATRVGSLGW, (iii) OmpC_220-237_: GGLKYDANNIYLAAQYTQ, (iv) OmpC_237-258_: TYNATRVGSLGWANKAQN.

Attenuated Total Reflection Fourier-Transform Infrared (ATR-FTIR) spectroscopy and Synchrotron Radiation Circular Dichroism (SRCD) measurements were performed as previously described in Turbant et al. [62]. Atomic Force Microscopy (AFM) experiments were conducted according to Wien et al. [63].

## 3. Results

The pH-dependent accumulation of ciprofloxacin in *E. coli* raises fundamental questions about how drug physicochemical properties, membrane permeability, and cellular regulation jointly determine fluoroquinolone uptake. To what extent is intracellular accumulation governed by CIP protonation equilibria and intrinsic physicochemical properties, and to what extent does it depend on regulated bacterial processes? Although OmpF and OmpC are established diffusion routes for hydrophilic antibiotics, pH-dependent accumulation persists in their absence, raising the question of which alternative envelope- or transport-associated mechanisms contribute to CIP uptake. How do Hfq-dependent regulatory pathways and proton homeostasis influence this process, and do their effects primarily reflect changes in membrane permeability or indirect remodeling of envelope composition and transport activity? Finally, how does intracellular pH, by altering the charge state of CIP, affect its membrane partitioning and intracellular retention? More broadly, how do drug physicochemistry and bacterial regulatory networks converge to determine fluoroquinolone accumulation in *E. coli*? These questions are addressed in this study.

### 3.1. Effect of Hfq Deletion on Ciprofloxacin Accumulation at Different pH

First, we tested the effects of the complete absence of the Hfq protein (Δ*hfq* mutant) on CIP accumulation in *E. coli* cells at different pH using single-cell spectrofluorimetry. Unlike bulk measurements, single-cell fluorescence enables real-time monitoring of CIP dynamics in individual intact cells, capturing the substantial heterogeneity known to exist within clonal bacterial populations [64,65,66]. Such heterogeneity, stemming from stochastic phenotypic switching, contributes to variable antibiotic resistance even in genetically identical bacteria. Moreover, intrinsic physicochemical properties of CIP, particularly its flexibility and protonation state, further modulate its accumulation on a cell-to-cell basis [67,68]. In order to assess phenotypic variability, at least 20-30 cells were analyzed in each experimental setup.

Three pH levels were tested: pH 5 to observe the accumulation of the cationic form of CIP, pH 7 for the zwitterionic form and pH 9 for the anionic form. While *E. coli* primarily inhabits the lower intestine, where pH rises from about 6 in the duodenum to 7–8 in the small intestine and colon [69], this bacterium may also survive temporarily in more extreme pH, such as in mildly acidic environments around pH 5 and mildly alkaline conditions around pH 9. These environments include biofilms, fermenting foods, soils, wastewater, parts of the digestive tract or dental plaque after sugar exposure [70]. Experiments performed at pH 9 also served as a platform to study the anionic form of CIP and porin-mediated uptake under alkaline conditions.

**Figure 1:**
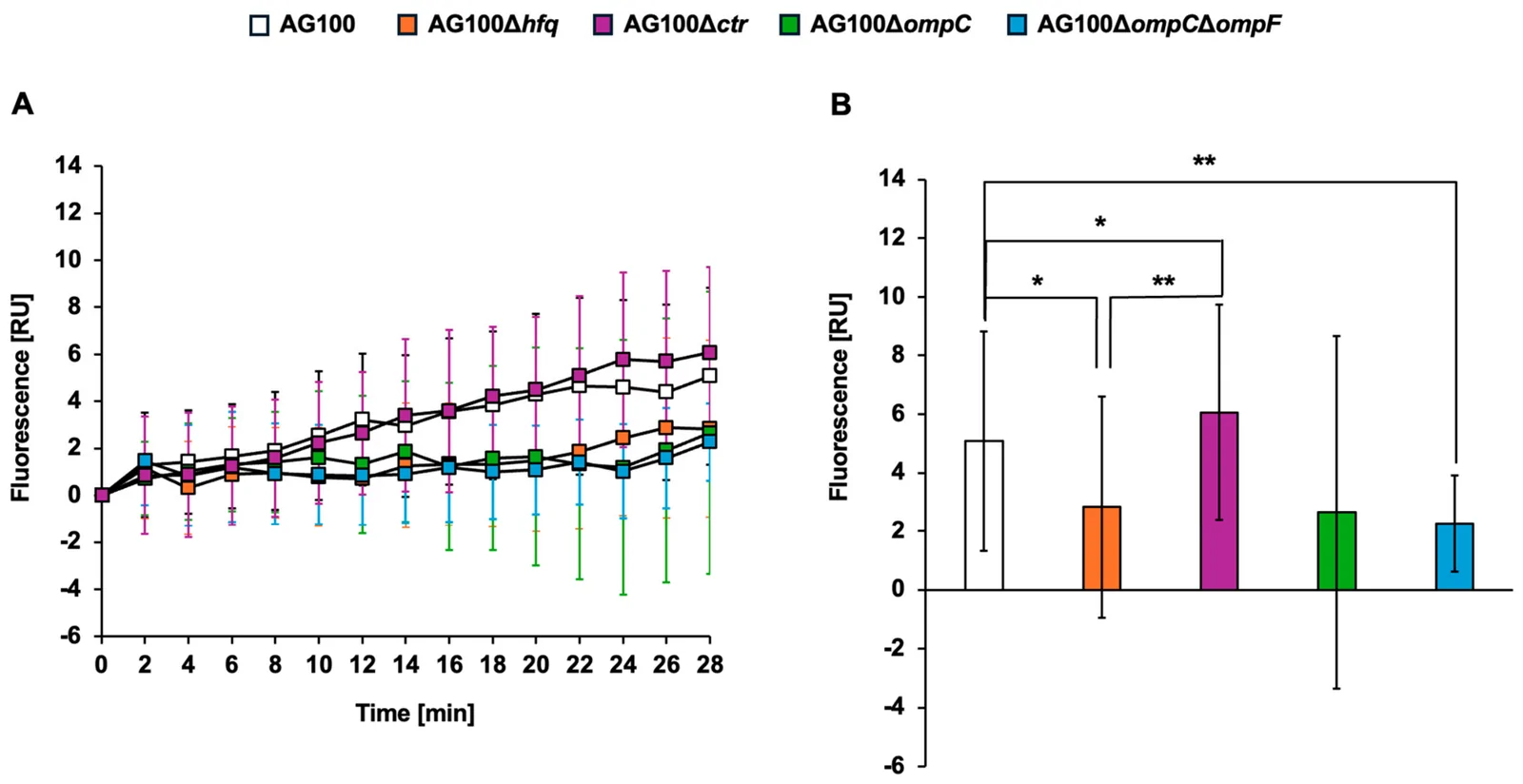
Kinetics of ciprofloxacin (CIP) accumulation at the single-cell level for AG100-derived bacteria measured for 28 min (**A**) and during the last time point (28 min) of the experiment (**B**) at pH 5. Results are shown as mean values from 3 biological replicates, each with at least 30 bacterial cells, pooled and analyzed together, with error bars indicating SD. Fluorescence emission is expressed in relative units (RU). White AG100 WT, orange AG100 ∆*hfq*, green AG100 Δ*ompC*, cyan AG100 Δ*ompC* Δ*ompF*, purple AG100 Δ*ctr*. The one-way ANOVA test with Tukey’s multiple comparison test was used to compare the means between tested strains at the last recorded time point (**B**). The significance of differences between compared experimental variants is marked by asterisks: *p* < 0.05 (*) or *p* < 0.01 (**). Bottom: An example of time course accumulation in live intact *E. coli* bacteria (here AG100 and AG100 Δ*hfq* strains at pH 5 from 0 to 28 min). Note that as Hfq regulates the expression of various morphogens, including MreB and BolA [71], and an apparent atypical morphology can sometimes be observed for some ∆*hfq* cells. The *hfq* deletion may additionally affect cell viability in the presence of CIP, as reported previously [26,49].

At pH 5, ∆*ompC*, ∆*ompC* ∆*ompF*, and ∆*hfq* strains all exhibited similarly low CIP accumulation, consistent with the limited penetration of CIP through OmpC and OmpF porins and its negligible diffusion across the lipid bilayer under acidic conditions (Figure 1). Although the ∆*hfq* mutation is expected to increase *ompC* and *ompF* expression, it produced only a minor effect on CIP accumulation, in agreement with previous studies showing that OmpC and OmpF contribute little to CIP influx at pH 5 because of altered porin closing probability [14], and that their increased expression in a ∆*hfq* background does not significantly enhance CIP uptake.

**Figure 2:**
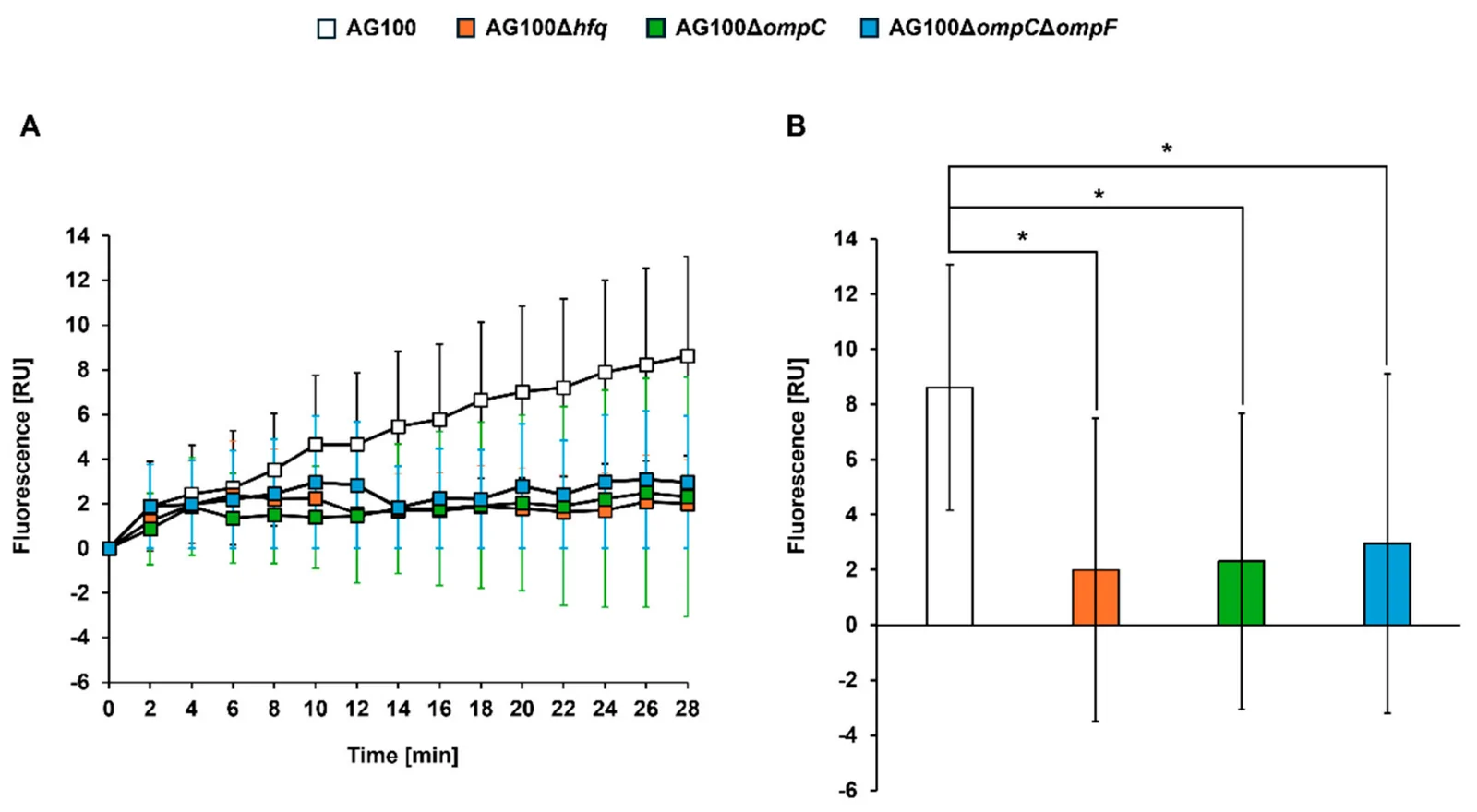
Kinetics of ciprofloxacin (CIP) accumulation at the single-cell level for AG100-derived bacteria measured for 28 min (**A**) and during the last time point (28 min) of the experiment (**B**) at pH 7. Results are shown as mean values from at least 20–30 bacteria analyzed for at least three different pre-cultures, with error bars indicating SD. White AG100 WT, orange AG100 ∆*hfq*, green AG100 Δ*ompC*, cyan AG100 Δ*ompC* Δ*ompF*. ANOVA test with Tukey’s multiple comparison test was used to compare the means between tested strains at the last recorded time point (**B**). The significance of differences between compared experimental variants is marked by asterisks: *p* < 0.05 (*).

At neutral pH 7, overall CIP accumulation increased relative to acidic conditions, as indicated by higher fluorescence intensities, consistent with enhanced CIP diffusion through the lipid bilayer; however, the relative trends among the mutants remained similar (Figure 2), in agreement with previous single-cell measurements in the same genetic background [26].

Note that in previous experiments, Δ*ompC* and Δ*ompF* mutants exhibited higher intracellular accumulation of CPI than the wild-type strain, suggesting that porins are not the sole pathway for antibiotic entry into bacterial cells (i.e., bilayer crossing). However, interpretation of these earlier findings was limited by substantial experimental variability, as reflected by the large error bars reported for both mutants [49]. In the present study, CIP accumulation measurements showed markedly reduced variability, due to improved pH control during the experiments. In the absence of OmpC and OmpF, CIP entry relies more heavily on porin-independent pathways, making accumulation particularly sensitive to extracellular pH fluctuations. The narrower error distributions observed herein are therefore consistent with the strong dependence of CIP membrane permeability on pH: even small deviations from neutral pH can alter its protonation state and consequently its membrane permeability [26,49]. Under the controlled neutral pH conditions used herein, the AG100 wild-type strain accumulated more CIP than the Δ*ompC* and Δ*ompC* Δ*ompF* mutants. This observation agrees with the established mechanism of ciprofloxacin uptake at neutral pH, where transport occurs predominantly through outer membrane porins when these channels are present. Importantly, extracellular pH control was experimentally validated using BCECF-based measurements of the surrounding cellular environment (see Section 3.3).

**Figure 3:**
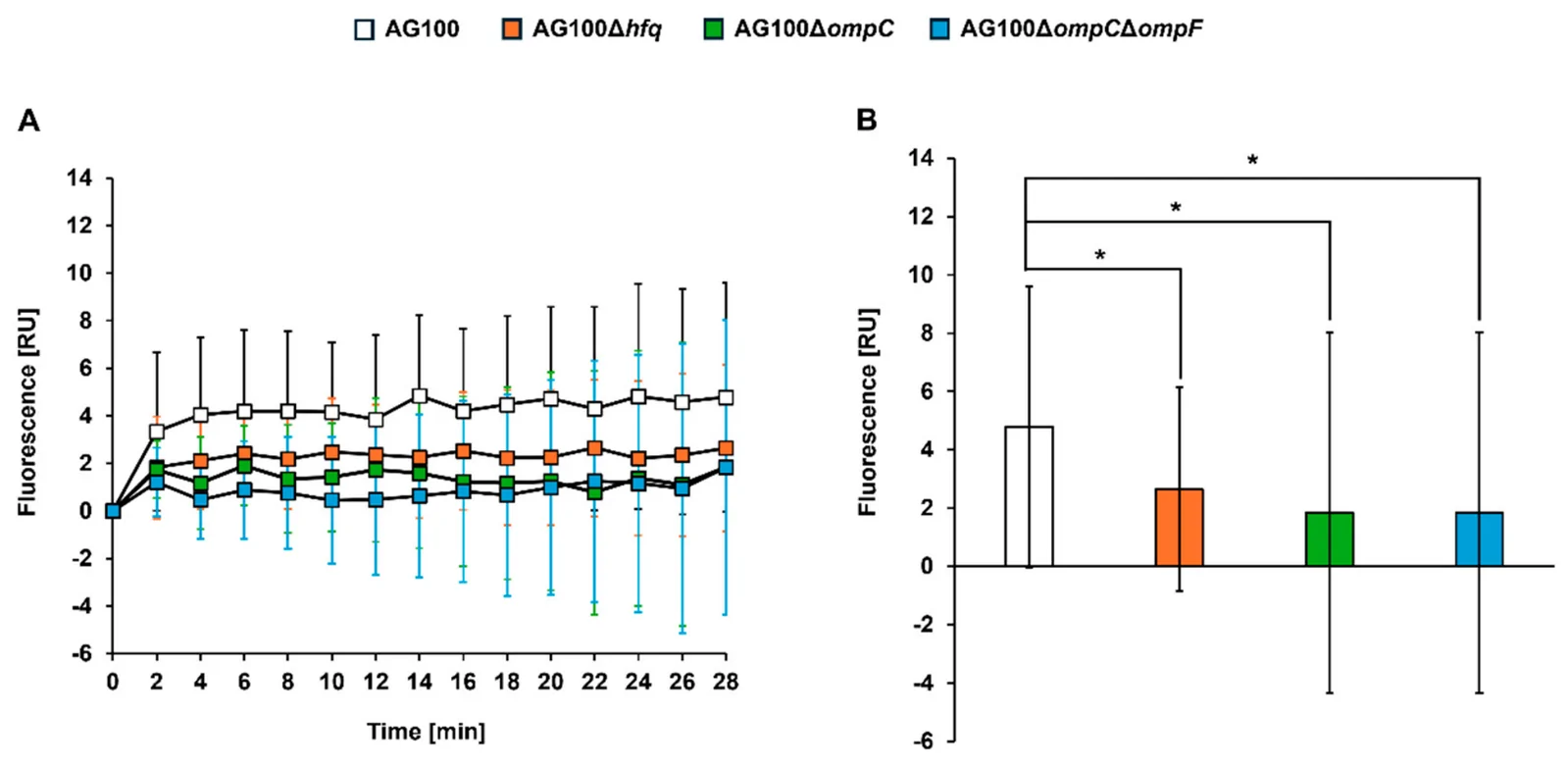
Kinetics of ciprofloxacin (CIP) accumulation at the single-cell level for AG100-derived bacteria measured for 28 min (**A**) and during the last time point (28 min) of the experiment (**B**) at pH 9. Results are shown as mean values from at least 20–30 bacteria analyzed for at least three different pre-cultures, with error bars indicating SD. White AG100 WT, orange AG100 ∆*hfq*, green AG100 Δ*ompC*, cyan AG100 Δ*ompC* Δ*ompF*. The one-way ANOVA test with Tukey’s multiple comparison test was used to compare the means between tested strains at the last recorded time point (**B**). The significance of differences between compared experimental variants is marked by asterisks: *p* < 0.05 (*).

At alkaline pH 9, CIP is negatively charged, limiting passive diffusion. Under these conditions, the CIP entry relies primarily on OmpC and OmpF porins, as reflected by low efficiency of its accumulation in the ∆*ompC* ∆*ompF* double mutant (Figure 3). Interestingly, the ∆*hfq* strain exhibited a slightly higher level of accumulated CIP relative to the ∆*ompC* single mutant and the ∆*ompC* ∆*ompF* double mutant; although this effect was moderate, it was close to the threshold for statistical significance, suggesting a trend rather than a major biological difference. Nevertheless, at high pH, Hfq is expected to act through sRNA-dependent repression of OmpC and OmpF porin-encoding genes, likely mediated by MicC and MicF. Because both sRNAs negatively regulate their target mRNAs encoding porins, the deletion of *hfq* should relieve this repression, increasing OmpC and OmpF porin levels, and consequently enhancing CIP uptake. We previously showed by RT-qPCR that, as expected, *hfq* deletion significantly increases *ompC* and *ompF* mRNA levels [49]. The observed decrease in uptake (Figure 3) therefore points to the existence of an additional regulatory mechanism. Note that ∆*ompC* ∆*ompF* mutants showed the lowest efficiency of the CIP import, while partial accumulation of this antibiotic was retained in the ∆*ompC* strain, a result in agreement with more efficient *ompF* expression relative to that of *ompC* at pH 9 [15,20,21].

An interesting observation emerged at pH 5, where both Omp-mediated transport and bilayer diffusion are expected to be negligible and therefore are unlikely to fully account for the observed CIP accumulation. Hfq-mediated membrane poration may contribute to CIP accumulation under these conditions [51,52]. To investigate this, we compared CIP accumulation at pH 5 across three strains: *hfq^+^* (WT), which is capable of membrane poration; ∆*hfq* and ∆*ctr*, both of which are deficient in this activity [51]. We observed increased CIP influx in ∆*ctr* compared to WT (Figure 1). This result aligns with our earlier observation at pH 7 [26]. However, the measurement at pH 5 suggests that the observed effect is not attributable to Hfq-mediated membrane poration, as Hfq in the absence of CTR is not capable of disrupting the membrane [51]. Instead, these results suggest that the CTR may exert negative effects on the expression of a gene coding for a putative protein involved in CIP import; such effects might be either direct or indirect.

### 3.2. Cell Growth and Viability of Different Mutants Under Various pH Conditions

**Figure 4:**
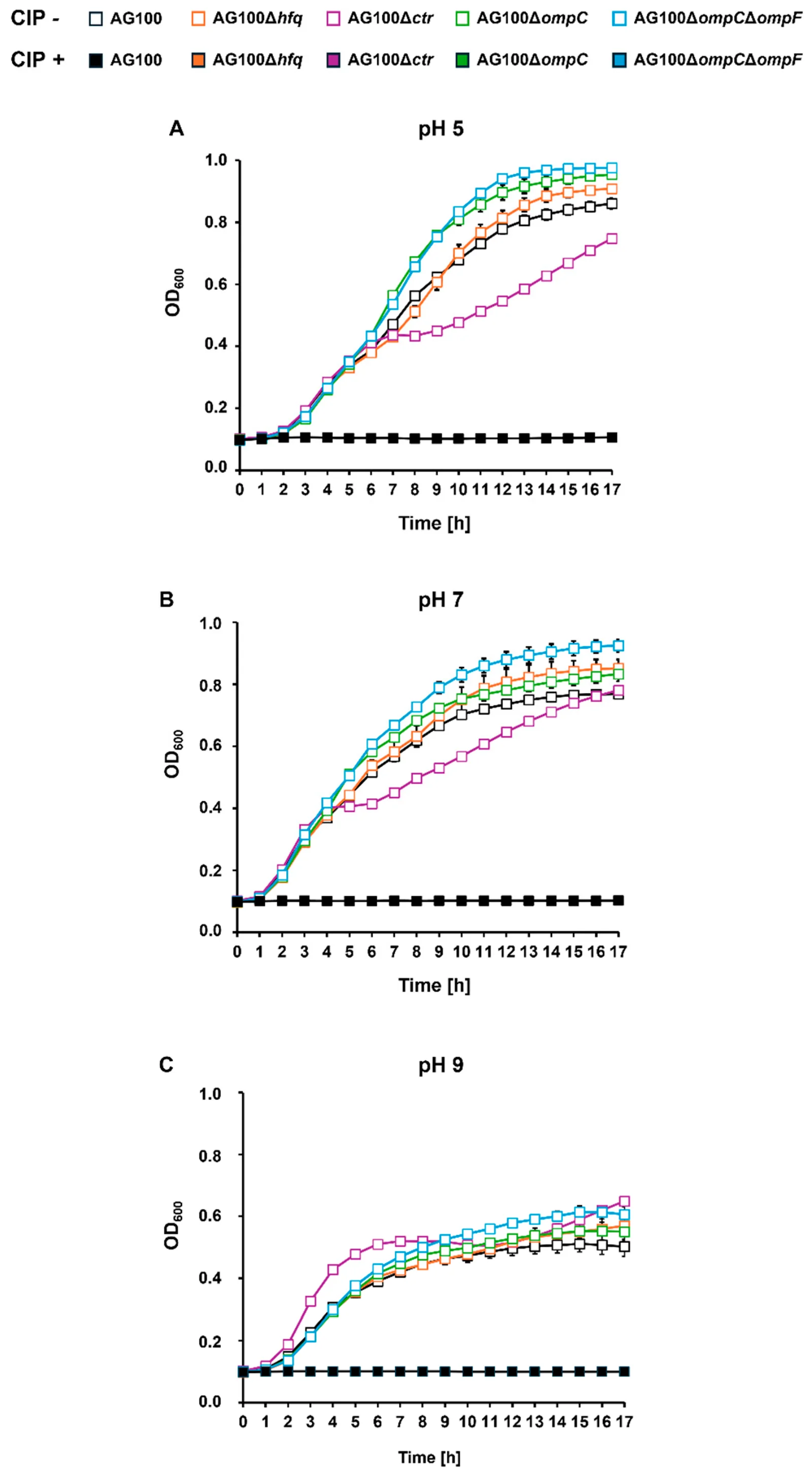
Kinetics of growth of *E. coli* AG100-derived bacteria in LB medium with different pH: 5 (**A**), 7 (**B**), and 9 (**C**) during incubation in the presence (filled squares) or absence (open squares) of CIP. The results are presented as OD_600_ of the bacterial cultures measured during a 17-h incubation at 37 °C. Mean values from three independent experiments are shown with error bars indicating SD. Note that in some cases the error bars are smaller than the symbols.

**Figure 5:**
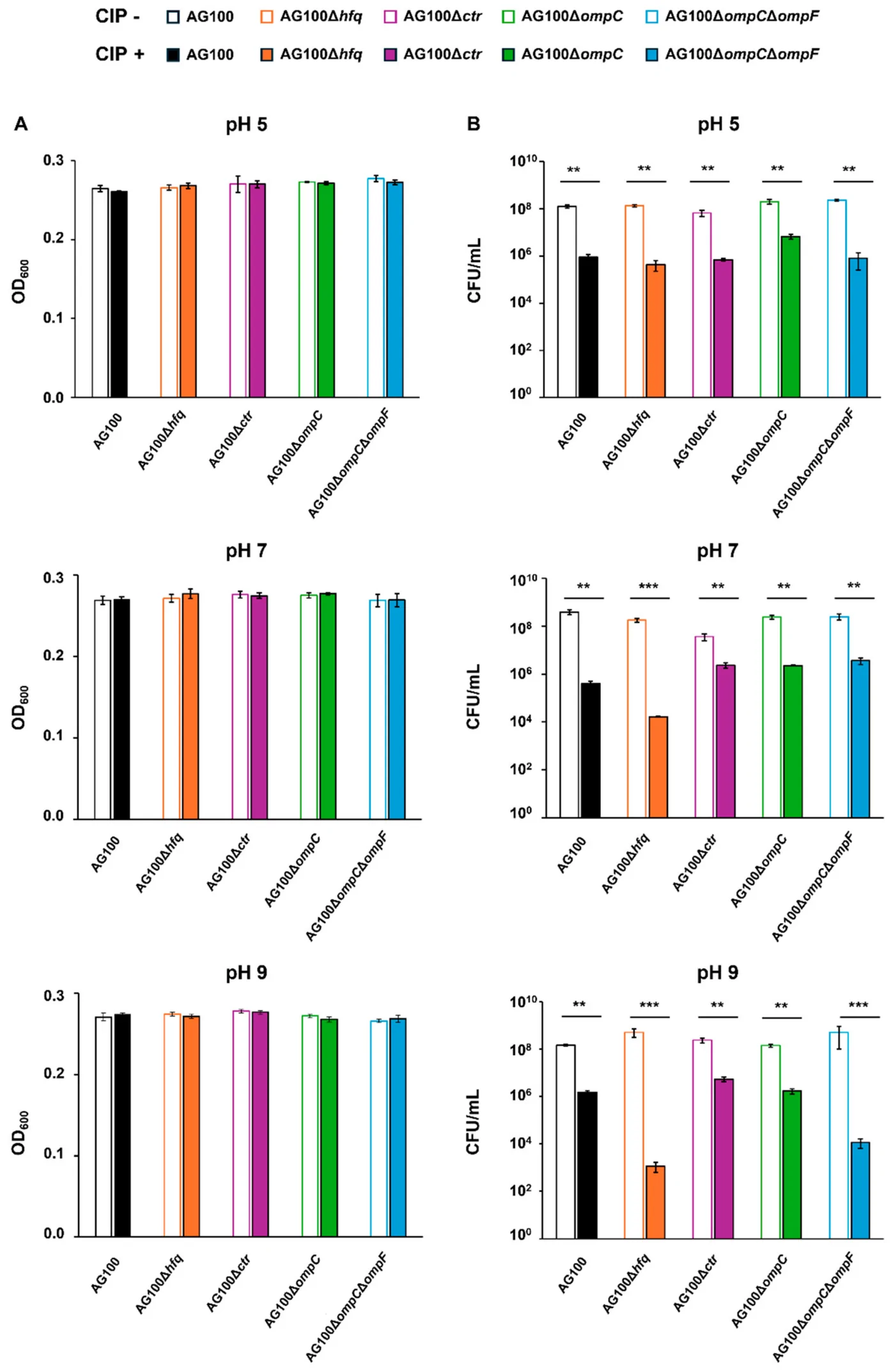
Effects of different pH (5, 7, and 9) and CIP accumulation on density (**A**) and viability (**B**) of *E. coli* AG100-derived bacteria culture. Results are shown as the bacterial culture density monitored as OD_600_ (**A**) and the number of colony-forming units per mL presented as CFU/mL (**B**) at the final time point (28 min) of the experiment. Bacteria were incubated in NaPi-Mg sodium phosphate buffer with pH 5, pH 7 or pH 9 in the presence (filled bars) or absence (open bars) of CIP. Mean values from three independent experiments are shown with error bars indicating SD. Statistical analysis was performed using a Student’s *t*-test. The significance of differences between compared experimental variants is marked by asterisks: *p* < 0.01 (**) or *p* < 0.001 (***).

Growth-rate measurements were conducted to test whether this parameter might influence the measurements described in previous sections. In the absence of CIP, with the exception of the AG100 Δ*ctr* strain, which exhibited slower growth, no significant differences in growth rates were observed among the other strains (Figure 4 and Figure 5). On the other hand, when CIP effects were monitored in long-time experiments, drastic inhibition of bacterial growth could be observed (Figure 4). Significant growth inhibition was also observed at 28 min time point after CIP addition (Figure 5).

### 3.3. Role of Hfq in Linking Intracellular pH Homeostasis to Ciprofloxacin Accumulation

**Figure 6:**
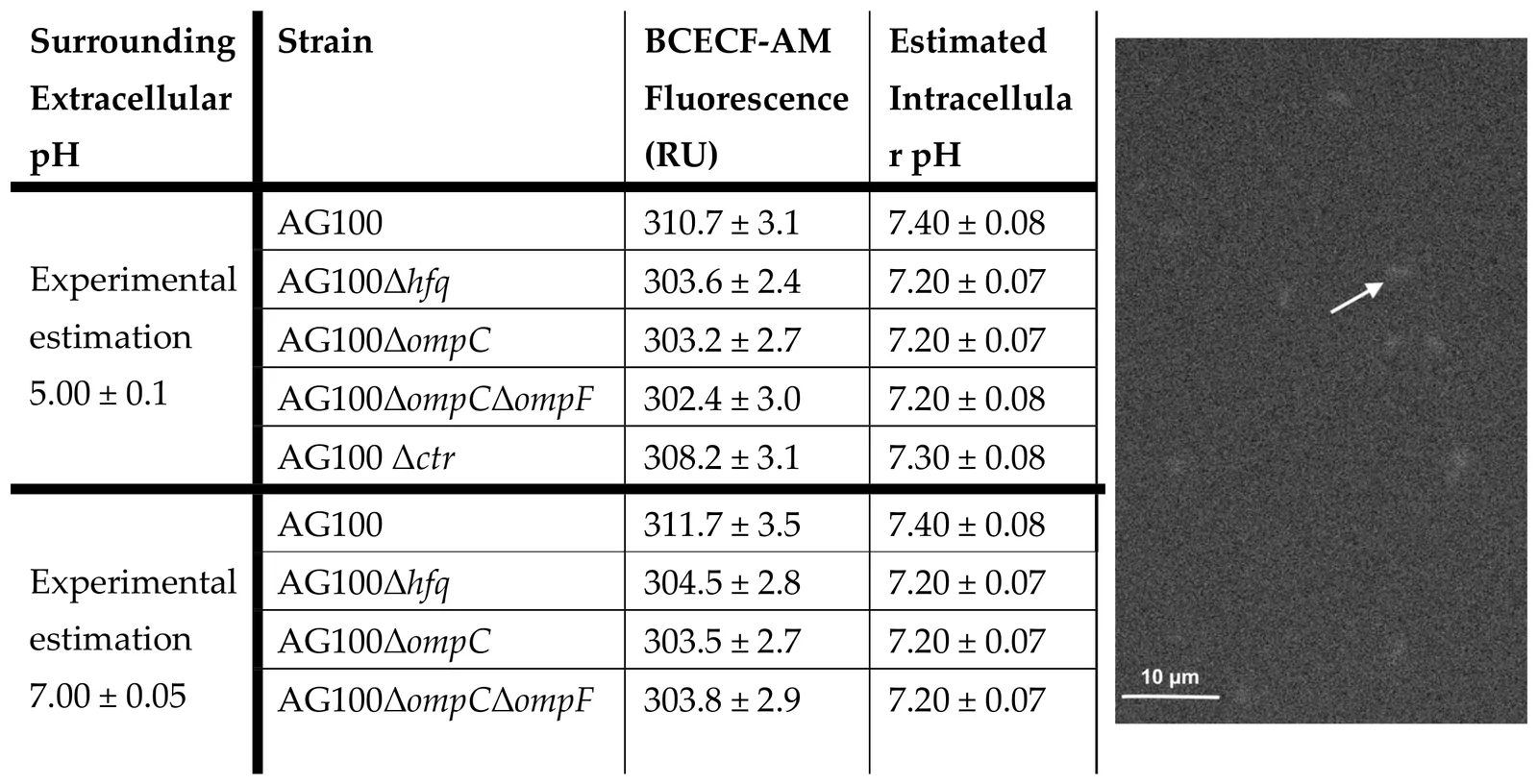
Single-cell pH estimation of wild-type (AG100) bacteria and isogenic Δ*hfq*, Δ*ompC*, Δ*ompC* Δ*ompF*, and Δ*ctr* mutants at pH 5 and pH 7. Due to the instability of BCECF-AM at basic pH, measurements could not be performed at pH 9. Results are shown as mean values from at least 20 bacteria analyzed from different pre-cultures (fluorescence emission is expressed in relative units RU). The right panel shows a representative example of AG100 Δ*hfq* bacteria analyzed at pH 5. A bacterium indicated by a white arrow is an example of cells exhibiting a fluorescence intensity of 303.2 RU. The extracellular pH was additionally assessed, confirming the stability of the controlled pH conditions throughout the experiments. All presented values measured for all mutants were statistically significantly different (*p* < 0.05) when compared to the wild-type (A100) counterpart.

An effect of Hfq on pH homeostasis may provide another explanation. To test this possibility, we used fluorescent probe BCECF-AM, which is well suited for probing cytosolic pH in *E. coli*, which typically maintains intracellular pH between 7.2 and 7.8, even under external acid or alkali stress [72]. BCECF-AM fluorescence increases with pH. Across pH 5 and 7 (corresponding experiments were not conducted at pH 9, because BCECF-AM is hydrolyzed under high pH values), we observed that WT cells maintained a higher cytoplasmic pH value, ~7.4 (in agreement with previously reported results [25]) when compared to ∆*hfq*, ∆*ompC*, and ∆*ompC* ∆*ompF* strains, which exhibited similar, lower pH values ~7.2 (Figure 6).

Deletion of *ompC* and/or *ompF* is not expected to directly affect intracellular pH, as these porins are not known to play a major role in H^+^ permeability [14]. The lower intracellular pH observed in these mutants might therefore arise indirectly from altered membrane permeability or associated changes in cellular physiology and growth. Similarly, the cytoplasmic acidification observed in the absence of Hfq could result from dysregulation of proton-consuming metabolic processes [60,73]. Interestingly, the Δ*ctr* strain exhibited an intermediate intracellular pH between those of the *hfq*^+^ and Δ*hfq* strains, suggesting that the Hfq-CTR might partially contribute to the maintenance of cytoplasmic pH.

Given the limited evidence for a contribution of Hfq-mediated membrane poration or permeabilization to CIP accumulation, we next explored whether Hfq might influence CIP uptake through the regulation of other porins. To test this hypothesis, we used three strains: *hfq*^+^, capable of sRNA regulation; ∆*hfq*, deficient in this function; and ∆*ctr*, retaining most sRNA-mediated regulatory activities. These measurements were performed at pH 5 where Omp-mediated transport is strongly reduced and passive bilayer diffusion is expected to be limited. At pH 5, ∆*ctr* showed a higher level of CIP accumulation than WT (Figure 1), suggesting that the Hfq-CTR may negatively regulate either the activity of a protein involved in CIP import or the expression of its gene. If so, the ∆*ctr* mutation relieves this inhibition/repression, enhancing CIP uptake. Although regulatory functions of the Hfq CTR appear to be relatively uncommon, they have been reported in certain contexts [41]. In particular, σE-regulated sRNAs are especially sensitive to deletion of this Hfq region [41,74]. One example is RybB, an sRNA that regulates multiple mRNAs encoding outer membrane proteins.

### 3.4. Potential Hfq-Driven Remodeling of Outer Membrane β-Barrel Proteins

Ultimately, although OmpC and OmpF are not expected to be major contributors to CIP influx at this pH, Hfq, particularly through its amyloidogenic CTR, might influence the structural state of outer membrane β-barrel proteins by promoting a transition from their native β-barrel conformation toward amyloid-like assemblies [75,76]. Hfq could modulate the equilibrium between these structural states and, consequently, alter OmpC permeability. The propensity of OmpC to adopt an amyloid-like conformation has previously been reported [77]. Our results provide further support for this proposal. Amyloid formation is typically characterized by a Circular Dichroism (CD) spectrum exhibiting a negative band near 220 nm, in contrast to the ~200 nm minimum observed for unfolded proteins [78]. Such a signature has previously been observed for OmpC as well [79]. These observations raise the possibility that Hfq could promote the conversion of OmpC into an amyloid-like assembly through cross-seeding or co-aggregation mechanisms [77]. Such conformational remodeling of the porin barrel could alter pore geometry, oligomerization, and electrostatic properties that could ultimately modify membrane permeability and substrate specificity, including antibiotic uptake.

**Figure 7:**
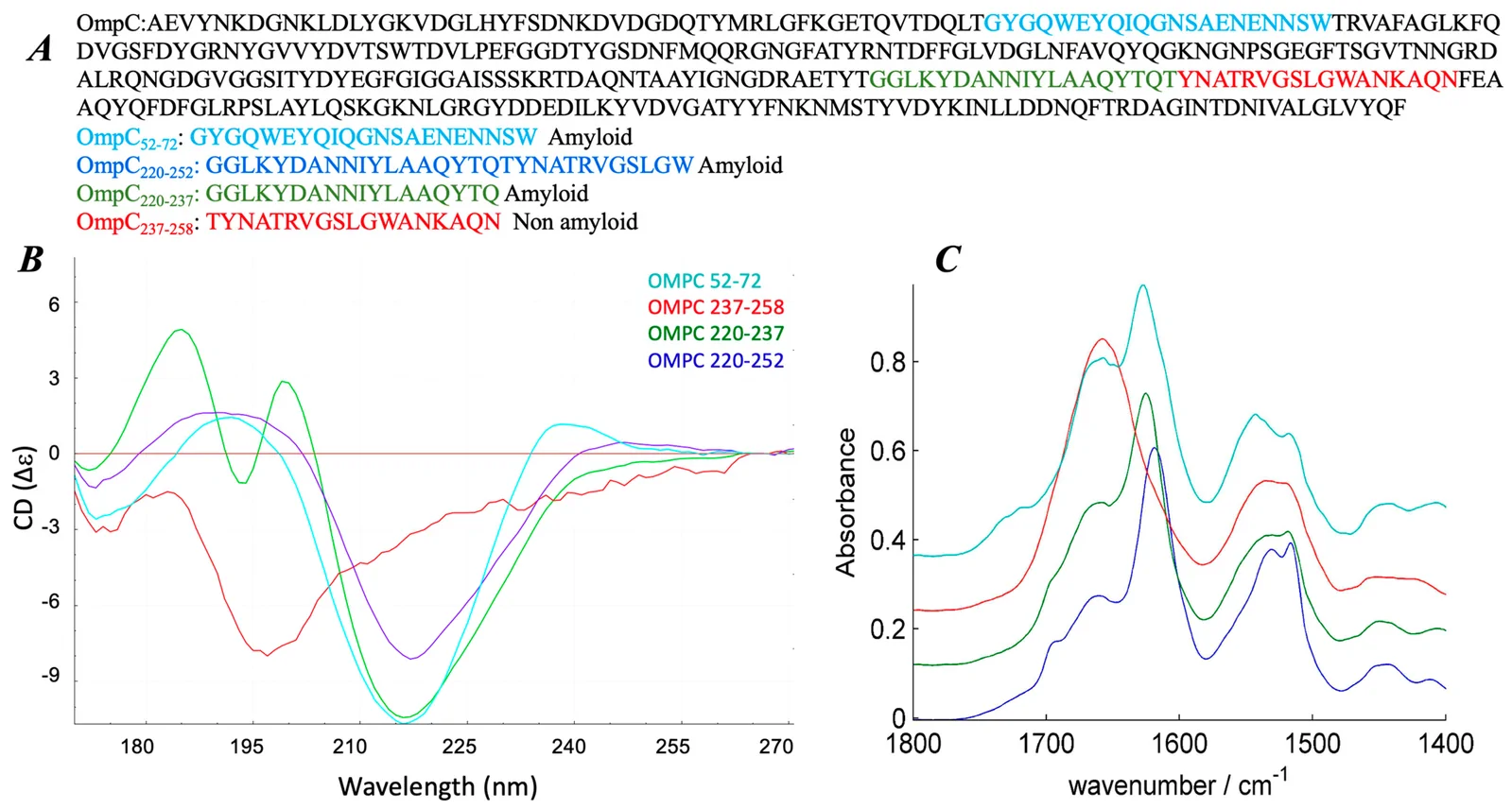
(**A**) Full-length OmpC sequence with potential amyloid regions cross-interacting with Hfq-CTR have been predicted using AmyloComp [80] and are highlighted in color; (**B**) SCRD spectra of OmpC peptides corresponding to potential amyloid regions; as a general guideline, a pronounced negative peak centered around 220 nm is indicative of amyloid structures, while the spectrum of an unfolded peptide shows a minimum near 200 nm [78]; (**C**) FTIR spectrum of the same peptides; the band near 1620 cm^−1^ is typically associated with amyloid β-sheet vibrations [81].

Putative cross-interacting regions between Hfq-CTR and OmpC were predicted using AmyloComp [80]. Four regions were identified: OmpC_52-72_ (GYGQWEYQIQGNSAENENNSW); OmpC_220-237_ (GGLKYDANNIYLAAQYTQ); OmpC_237-258_ (TYNATRVGSLGWANKAQN); OmpC_220-252_ (GGLKYDANNIYLAAQYTQTYNATRVGSLGW); note that OmpC_220-252_ combines OmpC_220-237_ and OmpC_237-258_. Among these peptides, only the latter three exhibited an amyloid spectral signature, based on Synchrotron Radiation Circular Dichroism (SRCD) and Fourier Transform Infrared (FTIR) spectroscopies (Figure 7) [78]. Notably, OmpC_220-237_ was particularly prone to amyloid formation.

**Figure 8:**
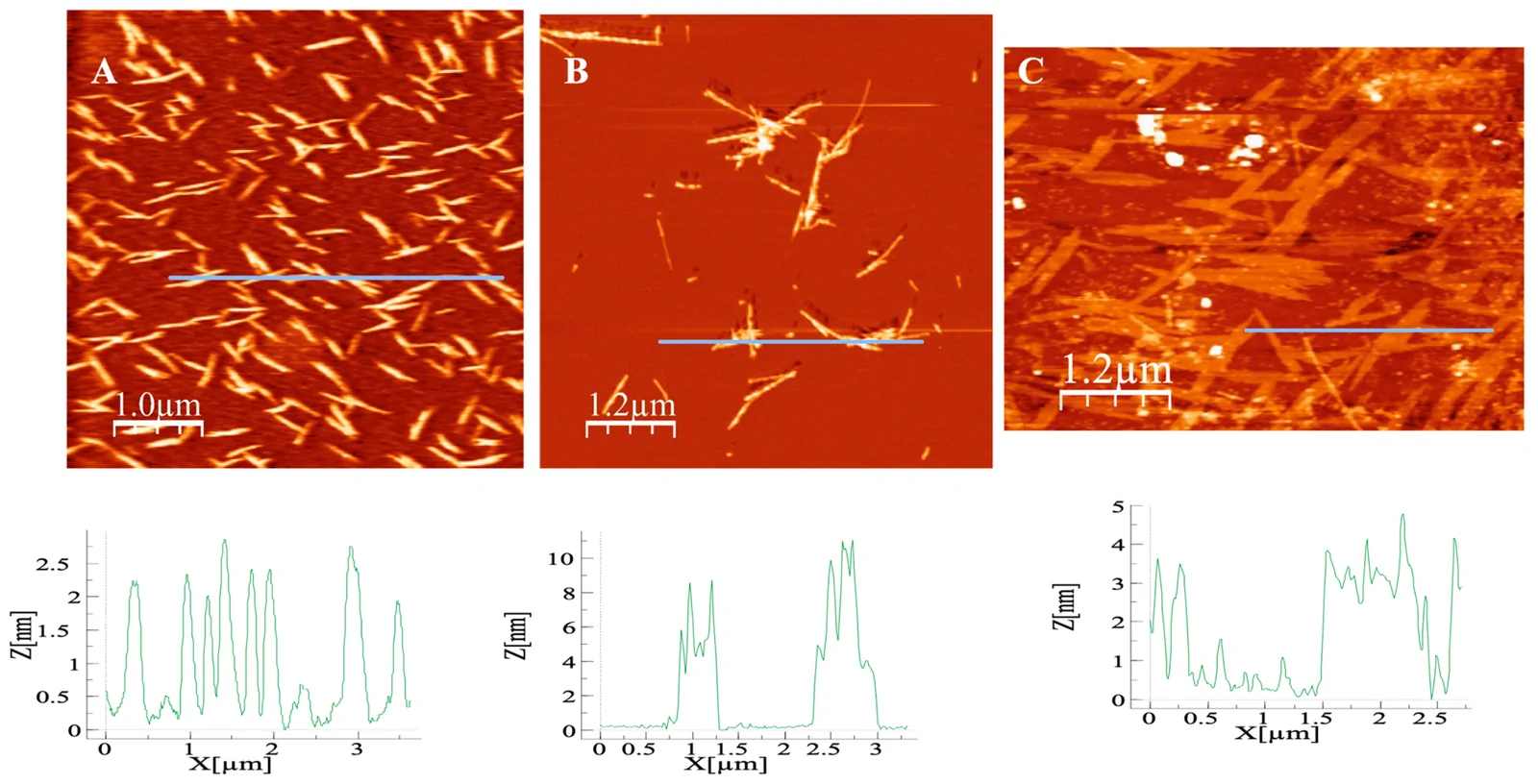
AFM images of individual pre-polymerized OmpC_220-237_ (**A**), Hfq-CTR (**B**) and of the mix of OmpC_220-237_ and Hfq-CTR after 16 h (**C**). Note that non-polymerized peptides such as OmpC_237-158_ cannot be observed using AFM, as their dimensions are below the typical resolution limit of the technique.

Based on in silico analyses predicting a more favorable interaction between the OmpC_220–237_ peptide and the Hfq CTR, together with an increased aggregation propensity [80], we further investigated the interaction between these peptides using molecular imaging. Atomic force microscopy (AFM) revealed that both the Hfq-CTR and OmpC_220–237_ formed fibrillar structures when examined separately (Figure 8A,B). Notably, when the two peptides were combined, the resulting structures displayed an architecture distinct from that observed for either peptide alone (Figure 8C), suggesting a structural interplay between the Hfq-CTR and OmpC_220–237_. Although these observations are consistent with a possible interaction between the two peptides, further experiments will be required to determine the nature and molecular basis of this interaction.

Although direct demonstration of an Hfq–OmpC interaction in vivo would further strengthen these findings, suitable fluorescent labeling strategies that preserve the native localization of Hfq are currently unavailable. In particular, C-terminal fluorescent fusions have been shown to impair Hfq activity and localization [82]. Interestingly, both Hfq and outer membrane porins have been reported to accumulate at the bacterial poles [83,84], providing a plausible spatial context for a potential interaction in vivo.

## 4. Discussion

The results presented here demonstrate that deletion of either full-length Hfq or its C-terminal region significantly affects ciprofloxacin accumulation in *E. coli*. Compared with the WT AG100 strain, Δ*hfq* and Δ*ompC* mutants exhibited reduced CIP accumulation across all three extracellular pH conditions tested (Figure 1, Figure 2 and Figure 3). We previously proposed that Hfq might enhance CIP accumulation independently of OmpC and OmpF by facilitating CIP passage through the lipid bilayer [49]. However, such a mechanism would be expected to depend strongly on CIP protonation state and membrane permeability. The observation that *hfq* deletion also affected CIP accumulation at pH 5, where passive bilayer diffusion of the predominantly cationic form of CIP is strongly limited, argues against a mechanism based solely on Hfq-mediated enhancement of lipid-mediated uptake. Moreover, Δ*hfq* cells exhibited reduced CIP accumulation at pH 7 and 9 (Figure 1 and Figure 3). These findings are unexpected because, under alkaline conditions, Hfq is generally expected to repress porin expression through MicC/MicF-dependent sRNA regulation [85]. Deletion of *hfq* should therefore relieve this repression, increase porin abundance, and potentially enhance CIP uptake. The observed phenotype consequently suggests that additional Hfq-dependent mechanisms counteract or outweigh the expected effect of increased porin expression. Importantly, strict control of extracellular pH is critical for interpreting these effects, particularly at pH 7, where even small deviations can alter CIP protonation and membrane permeability [26].

OmpC and OmpF are currently considered the major porins mediating fluoroquinolone uptake, whereas other outer membrane proteins have more specialized or indirect functions. LamB primarily facilitates maltodextrin uptake, OmpA contributes to envelope integrity, and *E. coli* lacks OmpD [86]. OmpX is involved in host–cell adhesion [87] and may indirectly contribute to antibiotic resistance through interactions with efflux systems [88]. Other porins, including PhoE (a general porin with a preference for phosphate-containing molecules), OmpW (a porin whose physiological substrate and function are not well characterized in *E. coli*), and the chitoporin ChiP, have been proposed as potential alternative routes for fluoroquinolone entry [12,89]. Among these, PhoE and ChiP have pore dimensions compatible with CIP diffusion, whereas OmpW forms a comparatively narrower channel. Direct experimental evidence for their contribution to CIP uptake remains lacking. However, our findings reinforce the hypothesis that Hfq influences CIP accumulation through mechanisms that may also involve other envelope-associated processes. Previous results might corroborate such a proposal. For example, in *Yersinia enterocolitica* the Hfq protein inhibits the expression of the gene encoding the OmpX protein [90]. In *E. coli*, double mutants in *hfq* and the *chiX* genes (revealing a compensatory phenotypical effect) were characterized by an increased efficiency of expression of the gene encoding the ChiP porin (though levels of mRNAs coding for OmpW and PhoE were unchanged relative to WT bacteria) [89]. Transcriptomic analyses provided evidence that removal of the CTR of Hfq resulted in a decrease in the levels of OmrA and OmrB (sRNAs responsible for reducing the expression of genes encoding outer membrane proteins, like ompT), ChiX (sRNA involved in reducing the expression of *chiP*), MicF (sRNA reducing the expression of *ompF*), and CyaR (sRNA reducing the expression of *ompX*) [41]. Finally, another transcriptomic study demonstrated that the Δ*hfq* mutation in *Klebsiella pneumoniae* resulted in both decreased efficiency of expression of the *micF* gene and impaired production of regulatory transcripts *omrA* and *omrB* [91]. Our results thus highlight the importance of Hfq and its CTR as elements that fine-tune multiple interconnected regulatory pathways.

Hfq and sRNAs may also regulate the expression of genes encoding membrane proteins that could influence how cells maintain intracellular pH homeostasis. The main defense of bacteria against cytoplasmic acidification is driven by proton-consuming reactions, e.g., those catalyzed by glutamate decarboxylases [60]. Hfq influences cytoplasmic pH by regulating the GAD system, which includes the glutamate decarboxylases GadA and GadB and the GadC glutamate/γ-aminobutyric acid (GABA) antiporter [92]. The Hfq-binding sRNA GadY, encoded in a σS-dependent manner in the *gadX-gadW* intergenic region, stabilizes *gadX* mRNA through base-pairing with its 3′-UTR [32]. GadX is a transcriptional activator of acid-resistance genes and promotes *gadABC* expression. In the absence of Hfq, GadY is unstable, leading to reduced GadX levels and poor GAD system functionality. As a result, glutamate decarboxylation, which normally consumes protons, is diminished, causing accumulation of cytosolic H^+^ and decreased intracellular pH. Furthermore, GadC functions as an antiporter, importing glutamate and exporting GABA. When GadC activity is lost, GadAB cannot access extracellular glutamate, halting proton-consuming decarboxylation reactions and further contributing to cytoplasmic acidification. This may explain why ∆*hfq* cells exhibit a lower intracellular pH compared with WT bacteria across all tested pH conditions. Note that the effect of Hfq on intracellular pH should influence only the cytoplasmic trapping of CIP, not its influx, which depends on extracellular pH [25]. In this context, the decrease in intracellular pH in the ∆*hfq* strain (to ~7.2), compared with a near-neutral periplasmic pH, may weaken and affect the cytoplasmic trapping effect of CIP, which becomes closer to neutral at pH 7.2 [25]. A potential side effect is that protonation of membrane lipid head groups at lower pH may reduce its anionic charge and/or the membrane integrity [93], thereby affecting CIP’s ability to diffuse across the lipid bilayer.

Ultimately, σ^S^, the alternative sigma factor that redirects RNA polymerase to specific gene sets during stationary phase and the general stress response, may also indirectly influence the expression of as-yet-unidentified CIP transport-associated factors or contribute to pH homeostasis [94]. In addition to MicC and MicF, several sRNAs participate in this regulatory network, notably through the regulation of *rpoS* and *hns* expression [16]. The expression of *rpoS* is regulated by DsrA, RprA, OxyS, and ArcZ, which collectively modulate stress-response pathways [95], while DsrA also regulates *hns* expression [96]. These interconnected regulatory pathways provide a potential framework through which Hfq-dependent sRNA networks could indirectly influence antibiotic accumulation. Notably, the Δ*ctr* strain is defective in DsrA-mediated stimulation of *rpoS* mRNA translation, which encodes σ^S^ [43,74].

Collectively, our findings indicate that Hfq, including its CTR, contributes to the regulation of CIP accumulation through mechanisms extending beyond its canonical role in sRNA-mediated regulation of *ompC* and *ompF*. Rather than acting through a single uptake pathway, Hfq may influence CIP accumulation through interconnected effects on porin regulation, membrane properties, and intracellular pH homeostasis. Our results further highlight the importance of rigorous control and real-time monitoring of pH in single-cell fluoroquinolone accumulation assays. CIP accumulation is a multifactorial process in which extracellular and intracellular pH, *hfq* allelic variation, membrane permeability, and the pH-dependent physicochemical properties of the antibiotic jointly determine intracellular drug levels. Changes in CIP ionization can alter membrane permeability and intracellular partitioning, while Hfq-dependent regulatory pathways may additionally modulate envelope composition, transport-associated functions, and pH homeostasis. Consequently, even small fluctuations in extracellular pH can introduce substantial variability and potentially obscure genotype-dependent differences in CIP accumulation.

It is important to indicate that the experimental system used in this work, as well as presented results, has some limitations. Since relatively high concentrations of CIP were used to obtain measurable effects in a short period of time, an antimicrobial effect and microbial stress conditions were evident, as demonstrated. Under such conditions, dormant cells could also form in the presence of CIP. Therefore, bacterial cell physiology was apparently affected significantly which should be considered when interpreting the results reported in this work. One might speculate that relatively big standard deviation values arose from those effects on cell physiology, causing huge variability from cell to cell. Because in our short-term experiments we did not distinguish between different forms of cells (physiologically active, dyeing, dead, dormant/persister cells), due to technical reasons, the differences in the physiology of those different cells could result in high intrapopulation variation in measured parameters, causing high SD values. A lack of genetic complementation experiments might also be considered a limitation of this study. However, while genetic complementation could confirm that ∆*hfq* phenotypes are linked to the mutation of interest, such an analysis is challenging because Hfq is a global regulator whose levels must be tightly controlled. Our attempts to restore the *hfq*-related phenotypes using a single-cell assay were unsuccessful, likely because plasmid-based expression of the *hfq* gene does not fully reproduce the native Hfq protein regulatory balance. This limitation reflects the complexity of ciprofloxacin accumulation phenotypes, which involve multiple interconnected regulatory pathways. Moreover, porin-level effects are inferred rather than directly measured in the present study, as changes in OmpC and OmpF abundance were assumed on the basis of previous transcriptomic analyses, not demonstrated as levels determined experimentally. Another limitation is the pH measurement method, where the setup did not allow precise ratiometric BCECF measurements and that the reported values should be treated as approximate pH estimates rather than accurate measurements. One should also note that direct in vivo demonstration of an Hfq–OmpC interaction is currently lacking. Therefore, Hfq peptide amyloid formation or co-aggregation does not prove full-length OmpC remodeling in cells, and the connection between these peptides and CIP uptake remains hypothetical. Finally, the lower intracellular pH observed in Δ*hfq* cells may represent an additional factor influencing the intracellular activity of CIP. As CIP is a zwitterionic molecule with pH-dependent ionization, the modest decrease in intracellular pH from approximately 7.4 to 7.2 could potentially alter its speciation and intracellular trapping. However, the magnitude of this pH shift cannot by itself establish a biologically meaningful change in CIP retention. Thus, altered CIP trapping due to the lower intracellular pH should be considered a possible contributing factor.

## 5. Conclusions

**Figure 9:**
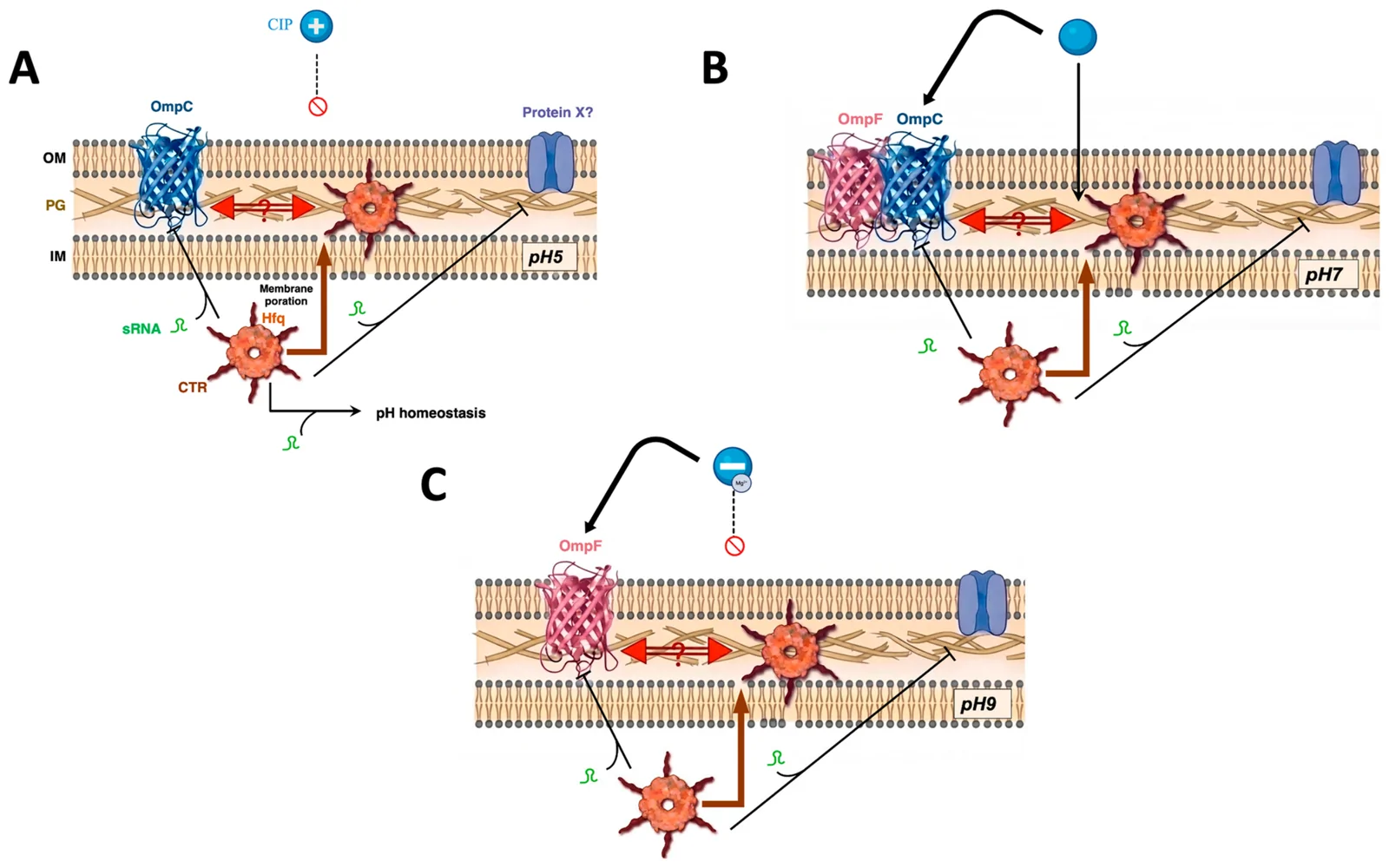
A hypothetical Hfq-dependent regulation influencing CIP fluxes. (**A**) At acidic pH, the positively charged fluoroquinolones (CIP+) are not expected to use the lipid-mediated pathway or the Omp porin-mediated pathway to cross the outer membrane (OmpC, depicted as a blue β-barrel, is the major porin at low pH). The *ompC* mRNA translation is directly repressed by MicC sRNA with the help of Hfq. Here we show that Hfq-CTR possibly negatively regulates a hypothetical protein involved in CIP import (called protein X herein, indicated with a question mark as a putative protein, depicted as light-purple protein in the OM) rather than acting through membrane poration. PhoE, OmpW, and ChiP porins, for instance, may be candidates to allow fluoroquinolone entry [12,89]. Note that Hfq and, in particular, its amyloid CTR could potentially compromise the shape of Omp β-barrels that could undergo a conformational switch from their native β-barrel architecture to an alternative amyloid form [75,76]. Hfq might therefore play a role in shifting the balance between these two structural states of the porins and change their selective permeability. (**B**) At pH 7 the neutral fluoroquinolone (CIP) uses both the lipid-mediated pathway and the OmpF porin-mediated pathway to cross the outer membrane. Translation of *ompF* and *ompC* mRNAs is repressed by MicF and MicC sRNAs, respectively, with the help of Hfq. (**C**) At pH 9 the negatively charged fluoroquinolones (CIP−) are not expected to use the lipid-mediated pathway but rather they could chelate Mg^2+^ (small periwinkle blue circle associated to CIP-) and use the OmpF porin-mediated pathway to cross the outer membrane. *ompF* mRNA translation is directly repressed by MicF sRNA with the help of Hfq. CIP is represented as a blue circle (CIP-, CIP or CIP+, depending on pH). Hfq-NTR is represented by an orange toroidal hexamer; Hfq-CTR by dark orange tails extending outside the NTR torus. sRNAs are depicted in green; OmpC, and OmpF are depicted as blue and pink β -barrels, respectively. Positive and negative regulation are indicated by black arrows and horizontal bars, respectively. The red arrow denotes the putative physical interaction between OmpC and Hfq.

This study suggests that CIP accumulation in *E. coli* results from the combined effects of multiple physicochemical and regulatory processes. Hfq might influence CIP accumulation through interconnected mechanisms involving outer membrane permeability, intracellular pH homeostasis, and the pH-dependent physicochemical properties of the antibiotic, while also contributing to cell-to-cell variability in accumulation. The reported results support the hypothesis about an Hfq-dependent pathway that contributes to CIP accumulation independently of the major porins OmpC and OmpF, although the molecular basis of this pathway remains to be determined. These results also suggest a possible involvement of an additional envelope-associated factor, but the identity and contribution of this factor remain to be determined. Overall, our findings suggest an integrated view of antibiotic accumulation in which bacterial regulatory networks interact with drug physicochemical properties to determine intracellular drug levels. They further highlight intracellular pH homeostasis as a possible determinant of the accumulation of weakly ionizable antibiotics. Accounting for both drug physicochemical properties and bacterial determinants of intracellular accumulation might thus provide new opportunities for the rational design and optimization of antibacterials against multidrug-resistant Gram-negative bacteria. A scheme depicting a speculative mechanism for Hfq-dependent regulation influencing CIP fluxes is presented in Figure 9.

## Figures and Tables

**Table 1:** *E. coli* strains used in this work.

Strain	Genotype	Reference
AG100	*argE3 thi-1 rpsL xyl mtl* Δ(*gal-uvrB*) *supE44*	[56]
AG100Δ*hfq*	Same as AG100 but Δ*hfq*::*cm*	[26]
AG100Δ*ctr*	Same as AG100 but *hfq*72-*cm* (∆*ctr*)	[26]
AG100Δ*ompC*	Same as AG100 but Δ*ompC*	[57]
AG100 Δ*ompC*Δ*ompF*	Same as AG100 but Δ*omp*C Δ*ompF*	[57]
AG100 Δ*ompC*Δ*ompF*Δ*hfq*	Same as AG100 but Δ*omp*C Δ*ompF* Δ*hfq*::*cm*	[49]

## Data Availability

The raw data supporting the conclusions of this article will be made available by the authors on request.

## Abbreviations

The following abbreviations are used in this manuscript:

CIP	Ciprofloxacin
OMP	Outer Membrane Protein
sRNA	small noncoding RNA
CTR/NTR	C-terminus/N-terminus region
BCECF(-AM)	Bis-CarboxyEthyl CarboxyFluorescein (acetoxymethyl ester)
SRCD	Synchrotron Radiation Circular Dichroism
FTIR	Fourier Transform Infrared
AFM	Atomic Force Microscopy
MDR	MultiDrug-Resistant
OM/IM	Outer and Inner Membranes
